# Current Treatment Approaches for Thymic Epithelial Tumors

**DOI:** 10.3390/life13051170

**Published:** 2023-05-12

**Authors:** Alfredo Tartarone, Rosa Lerose, Alessandro Rocco Lettini, Marina Tartarone

**Affiliations:** 1 Department of Onco-Hematology, Division of Medical Oncology, IRCCS-CROB Referral Cancer Center of Basilicata, 85028 Rionero in Vulture, Italy; 2 Hospital Pharmacy, IRCCS-CROB Referral Cancer Center of Basilicata, 85028 Rionero in Vulture, Italy; 3 Unit of Clinical Psychology, IRCCS-CROB Referral Cancer Center of Basilicata, 85028 Rionero in Vulture, Italy; 4 Faculty of Medicine, Humanitas University, 20090 Rozzano, Italy

**Keywords:** thymic epithelial tumors, thymoma, thymic carcinoma

## Abstract

Thymic epithelial tumors (TETs), including thymoma, thymic carcinoma and neuroendocrine tumors, are uncommon tumors that originate from the epithelial cells of the thymus. Nevertheless, despite their rarity, they represent the most common tumor type located in the anterior mediastinum. Therapeutic choices based on staging and histology may include surgery with or without neoadjuvant or adjuvant therapy represented by chemotherapy, radiotherapy or chemo-radiotherapy. For patients with advanced or metastatic TETs, platinum-based chemotherapy remains the standard first-line treatment; however, some new drugs and combinations are currently under evaluation. In any case, proper management of patients with TETs requires a multidisciplinary team approach to personalize care for each patient.

## 1. Introduction

Thymic epithelial tumors (TETs), including thymoma, thymic carcinoma and neuroendocrine tumors (NETs) are uncommon tumors that originate from the epithelial cells of the thymus. The crude incidence rate of TETs in Europe is 1.7 cases per million per year, the incidence rates are similar in both genders, and the mean age at diagnosis is 50–60 years [1,2], as indicated in Table 1. Thymomas, which account for about half of anterior mediastinal tumors, are more frequent than thymic carcinomas and NETs, which represent 14–22% and 2–5% of thymic epithelial neoplasms, respectively [3]. 

Thymoma and thymic carcinoma act differently considering that in thymoma the cancer cells look like the normal cells of the thymus in many ways, grow slowly and rarely spread beyond the thymus, while in thymic carcinoma the cancer cells do not look like the normal cells of the thymus, grow quickly and metastases spread early. The treatment algorithm for resectable thymic tumors includes surgery followed by further treatments (radiotherapy or chemotherapy) depending on radicality, histotype and stage, while for patients with advanced or metastatic TETs, platinum-based chemotherapy remains the standard first-line treatment. However, as we shall see, several new drugs such as the multikinase inhibitors lenvatinib and regorafenib, the chemotherapeutic agent S-1 (tegafur) and immune checkpoint inhibitors (ICIs) including pembrolizumab, avelumab and nivolumab are currently under evaluation regarding metastatic disease. In any case, proper management of patients with TETs requires a multidisciplinary team approach to personalize care for each patient.

Prognosis depends on various factors such as histology, stage and surgical radicality, with an overall 5-year survival rate of 78% for thymoma and 30% for thymic carcinoma [4]. In clinical practice, cases of thymomas are often found incidentally during diagnostic work-ups for myasthenia gravis (MG). In fact, TETs, particularly thymomas, are often associated with paraneoplastic autoimmune diseases (ADs) such as MG, which is observed in about 30% of patients. Other, less-frequent paraneoplastic ADs include pure red cell aplasia, hypogammaglobulinemia, polymyositis, Cushing syndrome and systemic lupus erythematous [5]. A recent paper published by Singhal et al. investigated the possible existence of risk factors, including molecular abnormalities, for ADs in 48 patients with advanced TETs treated at Stanford University until 2020 [5]. In particular, 16/48 patients (33%) had an associated paraneoplastic AD, mainly represented, as expected, by MG (44%). In this retrospective study the only characteristic associated with an increased risk of AD was a histology of thymoma. Considering the close relationship between TETs and paraneoplastic ADs, the ThYmic MalignanciEs (TYME) Italian collaborative group evaluated the safety of 245 doses of SARS-CoV-2 mRNA vaccine administered to 126 patients with TETs (30% affected by ADs) [6]. The authors recorded no G3–G4 toxicities and a rate of G1–2 adverse events (AEs) comparable with that reported in the general population. In the last few years in the scientific community, the interest in these rare tumors has increased, as demonstrated by the creation of several working groups, including the International Thymic Malignancy Interest Group (ITMG), the Reseau Tumeurs et THYMques et Cancer (RYTHMIC) network and the TYME Italian collaborative group.

In this paper we report the current management of patients with TETs with a special focus on novel medical therapies for the advanced disease.

## 2. Diagnostic Work-Up

The diagnostic work-up should include a careful medical history, physical examination (with particular attention to neurological signs), laboratory tests, comprising measurement of inflammatory markers and immunological tests, radiological exams and histopathological assessment. The differential diagnosis should be made with other solid tumors such as lymphomas (Hodgkin and non-Hodgkin lymphoma), extragonadal germ cell tumors and metastatic carcinomas that can also affect the mediastinum. Regarding radiological evaluation, a chest X-ray is often the first exam that suggests the presence of a thymic mass. Chest computed tomography (CT) scanning represents the imaging modality of choice for TETs, while magnetic resonance imaging (MRI) may be useful to evaluate cystic lesions or areas of local invasion [7]. Interestingly, Japanese researchers performed a CT-based radiomics analysis in 61 patients with TETs to distinguish between thymic carcinoma and thymoma [8]. They concluded that two texture features, gray-level co-occurrence matrix (GLCM)-energy and solidity, were significant predictors of thymic carcinoma. Araujo-Filho et al. showed that a CT-based radiomics model could also be effective in the preoperative prediction of resectability in patients with TETs [9]. Recently, other researchers retrospectively reviewed the CT findings and the prognoses of 194 patients with TETs (32 low-risk thymomas, 52 high-risk thymomas and 110 thymic carcinomas) [10]. The authors demonstrated that CT signs of vessel invasion and pericardial mass in patients with thymic carcinoma and pericardial mass in those with high-risk thymoma are related to poorer outcomes.

Overall, fluorodeoxyglucose (FDG) positron emission tomography (PET)/CT scanning does not yet have an established role in the evaluation of TETs, considering that it can provide false-positive results in case of infection, thymic hyperplasia and other non-neoplastic processes [2]. However, PET/CT scanning may be considered in the case of thymic carcinoma due to its higher tumor metabolism or for the detection of occult metastases [2]. The definite diagnosis is the histological one performed with the analysis of tissue samples derived from a biopsy in the case of advanced disease or directly from surgical specimen in the case of upfront operable disease. The preferred procedures used to obtain appropriate histological samples are percutaneous core-needle biopsy or incisional surgical biopsy through mini-thoracotomy or mediastinotomy, while fine-needle aspiration biopsy (FNAB) is generally not recommended [2]. In any case a high level of expertise is required from the pathologists. For example, a systematic review of 467 cases discussed by the RYTHMIC tumor board from January 2012 to December 2016 showed a discordance rate of 39%, including a 6% rate of major discordance [11]. Recently, researchers of the Mayo Clinic (Rochester, MN, USA) used a panel of CD117, BAP1, mTAP and TdT in a series of 81 TETs including 44 thymomas and 37 thymic carcinomas and concluded that this panel could be useful to distinguish thymomas from thymic carcinomas [12].

**Figure 1:**
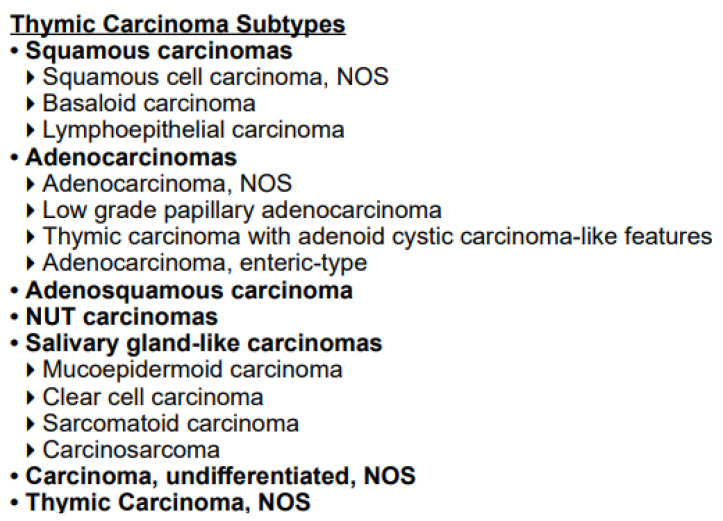
Histological classification for thymic carcinoma according to the World Health Organization (WHO) [13].

Table 2 and Figure 1 report the current available histologic classification for thymoma and thymic carcinoma according to the World Health Organization (WHO) [13]. In consonance with this classification, there are six entities represented by thymoma type A, AB, B1, B2, B3 and thymic carcinoma (type C). The subdivision into different subtypes is based on histologic characteristics including the content of non-neoplastic immature T-cells and neoplastic epithelial cells. Clinical aggressiveness increases in the following order: type A, AB, B1, B2, B3 and thymic carcinoma (type C). Thymic carcinoma includes several subtypes, the most common of which is squamous cell carcinoma.

**Figure 2:**
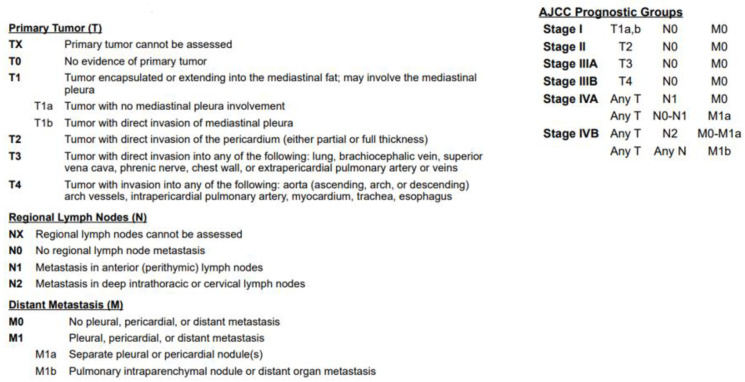
*TNM* 8th edition staging system for TETs [14].

**Figure 3:**
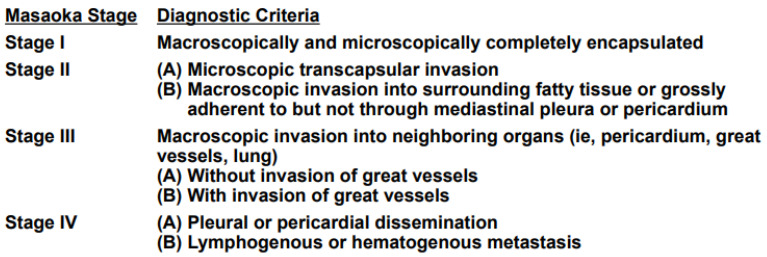
Modified Masaoka clinical staging system for thymoma [15].

Figure 2 and Figure 3 describe the *TNM* 8th edition system and the Masaoka–Koga staging system, which are the two main systems usually used in the classification of TETs [14,15]. 

The Masaoka–Koga staging classification includes four stages that have the following characteristics: stage I: intact thymic capsule; stage II: microscopic transcapsular invasion (A)/macroscopic invasion into adjacent mediastinal fat but not through mediastinal pleura or pericardium (B); stage III: macroscopic invasion into adjacent organs without invasion of great vessels (A)/with invasion of great vessels (B); and stage IV: pleural or pericardial dissemination (A)/distant metastases (B) [15]. It should be remembered that Masaoka–Koga staging is a surgical pathology system and that the use of TNM staging could be preferable in the event of the presence of lymph nodal and distant metastases.

## 3. Treatment

First of all, it is strongly recommended that the management of patients with TETs should be discussed by a multidisciplinary tumor board (MTB) involving not only medical oncologists, radiation oncologists and thoracic surgeons but also radiologists, neurologists, immunologists and pathologists. According to the European Society for Medical Oncology (ESMO) guidelines, the treatment algorithm for resectable thymic tumor (Masaoka–Koga stage I–III, TNM stage I–IIIA) includes upfront surgery followed by further treatments (radiotherapy or chemotherapy) depending on radicality (R1 vs. RO), histotype and stage [2]. Complete thymectomy represents the preferred surgical approach; however, if the tumor is widely invasive (stage III–IVA), en bloc resection of all involved structures should be carried out. Video-assisted thoracoscopic surgery (VATS) is an option for stage I–II tumors, while is not recommended for stage III tumors [2].

Chao et al. compared the outcomes of 140 patients with stage I–II thymoma who had undergone VATS or median sternotomy (MST) [16]. This study showed no statistically significant differences in five-year survival between the two study groups; however, VATS was associated with better perioperative outcomes. Lymphadenectomy N1 and N2 is recommended in the case of thymic carcinoma, considering the high rate of lymph node metastases related to this histology. Recently, Pastorino et al. reported the experience of an Italian referral center on a series of 644 patients with rare thoracic cancers, including 212 thymoma patients, undergoing a surgical procedure [17]. The surgical procedure complexity was classified as high, intermediate or low according to the extent of surgical resection and kind of associated reconstruction. In particular, infiltration of mediastinal great vessels (superior vena cava and innominate veins), common in advanced thymic neoplasms, increases the technical complexity to achieve complete resection at the vascular site and requires an effective reconstructive strategy. In this retrospective study, overall survival (OS) in patients with thymoma was 96.2% at 1 year, 84.8% at 5 years and 64.6% at 10 years.

The treatment algorithm for potentially resectable thymic tumor (Masaoka–Koga stage III–IVA) has as its first step the execution of a biopsy followed by primary chemotherapy; if the tumor becomes resectable, surgery followed by postoperative radiotherapy (45–50 Gy) is used, while if the tumor remains unresectable, definitive radiotherapy (60 Gy) or concurrent chemoradiotherapy (60 Gy, cisplatin and etoposide) is used [2]. Other available options in the case of Masaoka–Koga stage IVB include definitive concomitant chemoradiotherapy and definitive chemotherapy [2]. Regarding chemotherapy, up to six cycles of platinum–anthracycline-based regimens as CAP (cyclophosphamide, doxorubicin, cisplatin), a combination of carboplatin and paclitaxel, and cisplatin and etoposide represent the most effective options. A pooled analysis including 15 studies (10 prospective and 5 retrospective) indicates both that platinum with anthracycline-based chemotherapy is superior to platinum with non-anthracycline-based chemotherapy in terms of overall response rate (ORR 69.4% vs. 37.8%) in advanced thymoma and that cisplatin-based chemotherapy is superior to carboplatin-based chemotherapy (ORR 53.6% vs. 32.8%) in advanced thymic carcinoma [18]. 

Chinese researchers published a retrospective comparison of first-line platinum-based chemotherapy between a group of 36 patients with type B3 thymoma and a group of 127 patients with thymic carcinoma (including 64.6% with squamous carcinoma) [19]. Among all patients, there were not significant differences between B3 thymoma and thymic carcinoma in terms of PFS (11.3 vs. 10.1 months, *p* = 0.118) and OS (58.3 vs. 35.1 months, *p* = 0.067). PFS (11.3 vs. 11.7 months, *p* = 0.161) and OS (58.3 vs. 40 months, *p* = 0.114) did not differ between patients with B3 thymoma and those with squamous carcinoma, while OS was different between patients with B3 thymoma and those with non-squamous carcinoma (58.3 vs. 30.6 months, *p* = 0.031). The use of different therapy regimens did not result in any differences in terms of survival. The author concluded that both B3 thymoma and thymic carcinoma (especially squamous carcinoma) can benefit from first-line platinum-based chemotherapy.

Although there are no further recognized standard lines of chemotherapy, interesting results have been reported in the literature regarding the combination of capecitabine plus gemcitabine (CAP-GEM) and the tyrosine kinase inhibitor sunitinib, while imatinib can be considered for thymic carcinoma with c-KIT mutation [20,21,22,23]. A recent retrospective analysis from the TYME Italian collaborative group examined data from 20 patients with platinum-resistant TETs receiving continuous daily dosing (CDD) of sunitinib at a dosage of 37.5 mg [24]. The authors concluded that the CDD schedule showed similar effectiveness but a better toxicity profile as compared with intermittent dosing historical data.

## 4. New Therapeutic Options for the Treatment of Patients with Advanced TETs

Several advances, concerning in particular the treatment of patients with aggressive disease (thymoma B2–B3 and thymic carcinoma) in progression after at least one line of standard platinum-based chemotherapy, have been reported in the literature in the last few years.

In the Japanese phase II REMORA trial, 42 patients with advanced or metastatic thymic carcinoma that progressed after at least 1 platinum-based chemotherapy treatment received the novel multikinase inhibitor lenvatinib at a dosage of 24 mg orally once daily until disease progression or the occurrence of unacceptable toxicity [25]. The ORR, the primary endpoint of the study, was 38% with a disease control rate (DCR: complete or partial response plus stable disease) of 57% and a median duration of response of 11.6 months. The most common treatment-related adverse events (TRAEs) were hypertension, decreased platelet count, palmar-plantar erythrodysesthesia syndrome, decreased platelet count and diarrhea. Seven (16.7%) patients terminated the study due to TRAEs including intestine perforation, pneumothorax, arthralgia, left ventricular dysfunction and pneumonitis, but there were no toxic deaths. All patients had at least one-step dose reduction. The authors concluded that lenvatinib could become a standard second-line treatment option for patients with thymic carcinoma. In the Italian Resound trial, 19 patients with advanced or recurrent B2–B3 thymoma and thymic carcinoma previously treated with platinum-containing chemotherapy received the oral multikinase inhibitor regorafenib administered at a dose of 160 mg daily for 3 weeks of every 4-week cycle until disease progression or the development of unacceptable toxicity [26]. The authors reported partial response (PR) in 13 patients (68.4%) and stable disease (SD) in 2 patients (10.5%), evaluated according to the Choi criteria, which are based on a combination of the values of tumor size and tumor density obtained via CT [27], a median progression free survival (PFS) of 9.6 months and a median OS of 33.8 months. However, grade 3–4 TRAEs were observed in 10 patients (52.6%). 

Other Italian researchers evaluated the activity of everolimus in 51 patients with pretreated advanced thymoma (46% B2–B3) or thymic carcinoma [28]. They reported a DCR of 88%, a median PFS of 10.1 months and a median OS of 25.7 months. However, 14 patients (28%) had a serious TRAE, including liver toxicity, neutropenia and metabolic disorders, and 3 patients (6%) died due to pneumonitis.

Tsukita et al. evaluated S-1 (tegafur) in 40 previously treated patients with advanced thymoma and thymic carcinoma [29]. In this study the ORR was 17.5% (thymoma 10%; thymic carcinoma 25%), the median PFS was 7.0 months (thymoma 11.3 months; thymic carcinoma 5.4 months), and the median OS was 40.3 months (thymoma 58.5 months; thymic carcinoma 22.7 months). The main reported grade (G) 3–4 toxicities were anorexia (10%), neutropenia (7.5%) and pneumonitis (5%). 

Another similar Japanese study with S-1 (tegafur) administered to 26 previously treated patients with thymic carcinoma showed an ORR of 30.8%, a DCR of 80.8%, a median PFS of 4.3 months and a median OS of 27.4 months [30]. Rajan et al. evaluated the anti-programmed cell death ligand 1 (PD-L1) antibody avelumab, which is currently approved for the treatment of urothelial carcinoma and Merkel cell carcinoma, in a phase I trial including seven patients with advanced thymoma and one patient with thymic carcinoma treated with at least one prior standard therapy [31]. In this study two out seven patients with thymoma (29%) had PR, while three out eight patients (including one with thymic carcinoma) had SD (37.5%). The authors reported G ≥ 3 immune-related adverse events (irAEs) in five out of eight patients (62%). Cho et al. conducted a phase II study with the anti-programmed cell death 1 (PD-1) antibody pembrolizumab in 33 pre-treated patients with TETs (26 thymic carcinoma, 7 thymoma) and with no autoimmune disease to evaluate its safety and efficacy [32]. They reported in patients with thymoma 2 (28.6%) PR results and 5 (71.6%) SD results, while in patients with thymic carcinoma they reported 5 (19.2%) PR results and 14 (53.8%) SD results; the median PFS (mPFS) was 6.1 months for both thymoma and thymic carcinoma, while the median OS (mOS) was 14.5 months for thymic carcinoma and was not reached for thymoma. Regarding toxicity, 5/7 (71.4%) patients with thymoma and 4/26 (15.4%) with thymic carcinoma reported G ≥ 3 irAEs, including myocarditis, hepatitis, myasthenia gravis, colitis, thyroiditis and glomerulonephritis. Giaccone et al. administered pembrolizumab to 40 patients with recurrent thymic carcinoma who had progressed after at least one line of chemotherapy [33]. In this phase II study, the ORR was 22.5%, and the DCR was 75%. In a post hoc analysis, the PFS and OS were longer in patients with high PD-L1 expression than in those with low or no PD-L1 expression. However, although only patients with thymic carcinoma and without history of autoimmune disease were enrolled, six patients (15%) developed serious irAEs. In a small Japanese phase II trial named the PRIMER study conducted in the same patient population, the anti-PD-1 nivolumab did not achieve the same efficacy previously observed with pembrolizumab [34]. In fact, the only result was stable disease (SD) observed in 11 out of 15 patients that, however, lasted ≥ 24 weeks in 5 out 11 patients. In the same study 2 out of 15 patients experienced serious irAEs (grade II adrenal insufficiency and grade III aspartate aminotransferase increase).

A Chinese retrospective study that compared ICIs with chemo-immunotherapy (CT+ICIs) in 77 patients affected by thymic carcinoma (previous chemotherapy: 50 patients (64.9%), PD-L1 status unknown: 62 patients (80.5%)), showed better results for the combination CT+ICIs in terms of PFS (mPFS 12.7 vs. 2.1) [35]. No differences were observed in terms of OS (mOS 35.4 vs. 26.7 months); however, the mOS values in patients receiving ICIs or CT+ICIs as the second or further line of treatment were 26.7 and 45.4 months, respectively. The authors observed a high incidence (15.6%) of G3–4 irAEs, as we have seen in other studies conducted in this clinical setting with immunotherapy.

In the CAVEATT study 32 pre-treated patients (27 thymic carcinoma, 3 thymoma B3, 2 mixed-type thymic carcinoma + thymoma B3) with at least 1 line of platinum-based chemotherapy received a combination of the PD-L1 inhibitor avelumab plus the antiangiogenic agent axitinib [36]. The study showed the following results: an ORR of 34%, a DCR of 91% and an mOS of 26.6 months, with a 24-month OS of 52.2%. The most common G3–4 TRAE was hypertension (19%), while 4/32 patients (12%) developed serious irAEs including interstitial pneumonitis and polymyositis. In a small Chinese phase II trial, 25 pre-treated patients (13 with ≥ 2 prior therapy lines), 15 (60%) with thymic carcinoma and 10 (40%) with thymoma, were treated with the new oral antiangiogenic agent apatinib [37]. In this study the ORR was 40% and the DCR 84%, while the median PFS and the median OS were 9 and 24 months, respectively. With respect to toxicity, no G4 or 5 TRAEs were recorded, and, as expected, the most common G3 toxicity was hypertension (32%).

A Korean phase II study evaluated the role of the cyclin-dependent kinase (CDK) 4/6 inhibitor palbociclib in 48 patients with TETs (24 thymoma, 23 thymic carcinoma) who failed at least 1 line of chemotherapy [38]. The study showed the following results: an ORR of 12.5%, an mPFS of 11 months and an mOS of 26.4 months. The most common TRAEs were neutropenia, anemia and thrombocytopenia. The authors concluded that palbociclib might represent a salvage treatment for patients with TETs.

## 5. Immune Checkpoint Inhibitors in the Management of TETs: Pros and Cons

Immunotherapy with ICIs may be a potential option for the treatment of advanced refractory TETs, considering the high frequency of PD-L1 expression particularly in type B3 thymoma and thymic carcinoma and the lack of recognized effective therapies other than platinum-based chemotherapy [39].

In 2016 Yang et al. reported a case of metastatic thymic carcinoma which responded to anti-PD-1 therapy with pembrolizumab dramatically [40]. Subsequently, as previously shown, anti-PD-1 and anti-PD-L1 antibody treatment has been evaluated in several clinical trials in pre-treated patients [31,32,33,34,35,36]. Overall, ICIs with anti-PD-1 or anti-PD-L1 antibodies administered after the failure of standard chemotherapy are effective, with a clinical response in approximately 20% of cases; however, the treatment is associated with an enhanced risk of severe irAES, especially in the case of thymoma (up to 70% of G ≥ 3 irAEs), despite the exclusion in these trials of patients with active autoimmune disease. 

Reports of further cases of severe irAEs related to therapy with ICIs have been published recently. Liu et al. described 2 cases of severe immune myocarditis that occurred in patients with type B2 and B3 thymoma treated with gemcitabine, carboplatin and sintilimab (patient 1) or docetaxel, cisplatin and tislelizumab (patient 2) [41]. Patient 1 died within a few days. Mullenix et al. described three cases of ICIs inducing polymyalgia rheumatica (PMR)-like illness in patients with TETs enrolled in one of two clinical trials with avelumab or pembrolizumab (NCT 02146170, NCT 03076554) [42]. In contrast to myositis, which is characterized by elevated muscle enzymes and muscle weakness, PMR is characterized by morning stiffness and muscle pain. In addition, these patients should be monitored also for symptoms of giant cell arteritis including headache, double vision, flu-like symptoms and pain over the temples. Management of ICI-induced PMR-like illness required the use of corticosteroids and, in the case of sub-optimal response to steroids, anti-rheumatic drugs or anti-cytokine therapies such as tocilizumab. Jing et al. reported four cases of fatal toxicity caused by the anti-PD-1 inhibitors pembrolizumab and sintilimab regarding four patients with metastatic TETs who were ineligible for first-line platinum-based chemotherapy [43]. 

The combined use of anti-angiogenic therapy and ICIs could have synergistic activity, as reported by Conforti et al. in the CAVEATT study [36]. In fact, the authors reported interesting positive outcomes (an ORR of 34%, a DCR of 91%, a PFS of 7.5 months and a 24-month OS of 52.2%) with no drug-related deaths, although 4/32 patients (12%) developed serious irAEs.

The increased risk for irAEs in thymoma, as suggested by Ohm et al., could be explained by their immune microenvironment, including immature T cells that may provide the base for autoimmune reactions and therefore irAEs [44]. Thymic carcinoma and type B3 thymoma, which instead are characterized by an infiltration of mature T lymphocytes, consequently have lower risks of irAEs during ICI therapy and for this reason should be the two entities of choice regarding the use of these agents. He et al. analyzed the genomic profile of 10 patient samples (5 non-responders versus 5 responders) enrolled in their phase II study by using whole-transcriptome sequencing and whole-exome sequencing with the aim of identifying potential predictors of response to immunotherapy [45]. They found that alterations in genes that correlated with PD-L1 expression (BAP1 and CYLD) could be predictors for resistance response to immunotherapy. Recently, the NCCN guidelines added pembrolizumab as a possible therapeutic option for the treatment of refractory thymic carcinoma in view of its promising antitumor activity [46].

Table 3, which summarizes the main ongoing clinical trials in patients with advanced TETs, shows how immunotherapy administered alone or in combination with other agents is currently under study in several trials including patients with advanced B3 thymoma and thymic carcinoma which relapsed after at least one line of platinum-based chemotherapy. Interestingly, an ongoing Korean phase II study (NCT 03858582) will evaluate the efficacy and safety of neoadjuvant treatment with pembrolizumab at a dose of 200 mg plus chemotherapy (docetaxel 75 mg/m^2^ + cisplatin 75 mg/m^2^) for 3 cycles every 3 weeks. Patients with R0 resection will receive pembrolizumab at a dose of 200 mg for 32 cycles, while patients who had R1 or R2 resection will receive radiation therapy and pembrolizumab at a dose of 200 mg for 32 cycles. 

## 6. Conclusions

TETs are rare and heterogeneous tumors. Their diagnosis is often incidental during a diagnostic work-up for MG because they, especially thymomas, are often associated with paraneoplastic Ads due to alterations in self-tolerance and the expression of novel antigens. The management of TETS requires a multidisciplinary approach in referral centers, considering the risk for the physicians to make a late or incorrect diagnosis and the need for the patients to start early the appropriate therapies. The creation of virtual MTB involving experts (pathologists, oncologists, radiation oncologists and thoracic surgeons) from geographically distant institutions might be a useful tool, as it is for the management of other rare cancers. For example, in France within the RYTHMIC network, the treatment of patients with TETs is discussed at a national MTB which is organized using a web-based system. Usually, surgery is the cornerstone of the treatment of these neoplasms when they are diagnosed in early stages, while platinum-based chemotherapy is the treatment of choice in case of metastatic disease. However, patients with metastatic TETs have limited treatment options beyond platinum-based chemotherapy due to the poor effectiveness showed by several other agents administered in subsequent lines of therapy, and for this reason new therapies have been explored in this clinical setting such as the antiangiogenic multikinase inhibitors lenvatinib and regorafenib, the mTOR inhibitor everolimus, the chemotherapeutic agent S-1 (tegafur) and ICIs. As we have previously reported, there are promising data on the use of anti-PD-1 and anti-PD-L1 antibodies including pembrolizumab, avelumab and nivolumab, but immune-related toxicity should be kept in mind. In fact, given the high incidence of autoimmunity, additional studies are needed to identify those who can benefit from ICIs without irAEs or with acceptable irAEs (<grade 3 toxicities). For these reasons, as reported in Table 3, ICIs are under evaluation in several ongoing studies including patients with thymic carcinoma and type B3 thymoma who have a lower risk of irAEs.

## Figures and Tables

**Table 1:** Epidemiology of TETs (thymic epithelial tumors).

Heterogeneous Group of Malignancies
Incidence: 1.7/1 million/year
Most common tumor type located in the anterior mediastinum
Any age (mean age at diagnosis 50–60 years), both genders (slight male prevalence)
No risk factors identified
Common association with autoimmune diseases (myasthenia gravis in 30% of the cases)
Prognosis: 5-year survival ~80% for thymoma, ~30% for thymic carcinoma

**Table 2:** Histological classification for thymoma according to the World Health Organization (WHO) [13].

Thymoma Subtypes	Obligatory Criteria	Optional Criteria
Type A	Occurence of bland, spindle shaped epithelial cells; paucity or absence of immature T cells	Polygonal epithelial cells CD20+ Epithelial cells
Atypical type A variant	Criteria of type A, in addition comedo-type tumor necrosis; increased mitotic count, nuclear crowding	Polygonal epithelial cells CD20+ Epithelial cells
Type AB	Occurrence of bland, spindle shaped epithelial cells; abundance of immature T cells	Polygonal epithelial cells CD20+ Epithelial cells
Type B1	Thymus-like architecture and cytology; abundance of immature T cells, areas of medullary differentiation; paucity of polygonal or dendritic epithelia cells without clustering	Hassall’s corpuscles; perivascular spaces
Type B2	Increased numbers of single or clustered polygonal or dendritic epithelial cells intermingled with abundant immature T cells	Medullary islands; Hassall’s corpuscles; perivascular spaces
Type B3	Sheets of polygonal slightly to moderately atypical epithelial cells; absent or rare intercellular bridges; paucity or absence of intermingled T cells	Hassall’s corpuscles; perivascular spaces
MNT (micronodular thymoma with lymphoid stroma)	Nodules of bland spindle or oval epithelial cells surrounded by an epithelial cell-free lymphoid strome	Lymphoid follicles; monoclonal B cells and/or plasma cells
Metaplastic thymoma	Biphasic tumor composed of solid areas of epithelial cells in a background of bland-looking spindle cells; absence of immature T cells	Pleomorphism of epithelial cells; actin, keratin, or EMA-positive spindle cells
Rare others (microscopic thymoma, sclerosing thymoma, lipofibroadenoma)		

**Table 3:** Ongoing clinical trials in patients with TETs [47].

Study Title	ClinicalTrials.gov Identifier	Patient Population	Phase	Drug	Primary Endpoint	Country
Nivolumab in patients with type B3 T and TC (NIVOTHYM)	NCT03134118	Advanced B3 T and TC relapsed after at least one line of P-CHT	II	Nivolumab	PFS	Several European states
A pilot study to investigate the safety and clinical activity of avelumab in T and TC after progression on platinum-based chemotherapy	NCT03076554	Advanced T and TC relapsed after at least one line of P-CHT	II	Avelumab	Safety ORR	United States
Bintrafusp alfa (M7824) in subjects with T and TC	NCT04417660	Advanced T and TC relapsed after at least one line of P-CHT	II	Bintrafusp alfa (M7824)	ORR	United States
Trial of sunitinib in patients with type B3 T or TC in second and further lines (Style Trial)	NCT03449173	Advanced B3 T and TC relapsed after at least one line of P-CHT	II	Sunitinib	ORR	Italy
PT-112 in subjects with T and TC	NCT05104736	Advanced T and TC relapsed after at least one line of P-CHT	II	PT-112	ORR	United States
A study of KC1036 in patients with advanced TC	NCT05683886	Advanced recurrent, unresectable and/or metastatic T	II	KC1036	ORR	China
Pembrolizumab in treating participants with unresectable T or TC	NCT03295227	Unresectable T or TC	I	Pembrolizumab	Safety	United States
Combination of pembrolizumab and lenvatinib in pre-treated TC patients (PECATI)	NCT04710628	Advanced B3 T and TC relapsed after at least one line of P-CHT	II	Pembrolizumab Lenvatinib	PFS	Several European states
A study of KN046 in patients with TC who failed ICIs	NCT04925947	Advanced TC relapsed after P-CHT and at least one line of ICIs	II	KN046	ORR	United States
KN046 in subjects with TC	NCT04469725	Advanced TC relapsed after at least one line of P-CHT	II	KN046	ORR	China
Pembrolizumab and sunitinib malate in treating participants with refractory metastatic or unresectable TC	NCT03463460	Advanced TC relapsed after at least one line of P-CHT	II	Pembrolizumab Sunitinib	ORR	United States
Carboplatin and paclitaxel with or without ramucirumab in treating patients with locally advanced, recurrent or metastatic TC	NCT03694002	Advanced TC with no anti-cancer therapy for locally advanced or metastatic disease	II	Carboplatin Paclitaxel Ramucirumab	PFS	United States
Ramucirumab and carbo-paclitaxel for untreated thymic carcinoma/B3 thymoma with carcinoma RELEVENT Trial	NCT03921671	Chemotherapy-naïve patients with thymic carcinoma or B3 thymoma with areas of carcinoma	II	Carboplatin Paclitaxel Ramucirumab	ORR	Italy
A Phase II, neo-adjuvant pembrolizumab, docetaxel, cisplatin therapy followed by surgery and pembrolizumab consolidation therapy in locally advanced thymic epithelial tumor (TET)	NCT03858582	Locally advanced thymic epithelial tumor (TET)	II	Pembrolizumab Docetaxel Cisplatin	Major pathologic response rate	Korea

Legend: TETs: thymic epithelial tumors; T: thymoma; TC: thymic carcinoma; P-CHT: platinum-based chemotherapy; ICIs: immune checkpoint inhibitors; PFS: progression-free survival; ORR: overall response rate.

## Data Availability

Not applicable.
